# Paths out of poverty: Social entrepreneurship and sustainable development

**DOI:** 10.3389/fpsyg.2022.1062669

**Published:** 2022-11-30

**Authors:** Xiaoyi Zhang, Yu Sun, Yang Gao, Yueqi Dong

**Affiliations:** ^1^School of Biological and Agricultural Engineering, Jilin University, Changchun, China; ^2^School of Economics and Management, Dalian University of Technology, Dalian, China; ^3^Management School, Hainan University, Haikou, China

**Keywords:** rural poverty, social entrepreneurship, social opportunity, sustainability, case study

## Abstract

Poverty reduction in rural areas is an important development goal concerned by the international community, but the traditional poverty-reduction methods have certain drawbacks. Social entrepreneurship, with its innovative way to solve social problems, has gradually become a new sustainable development path to solve rural poverty. Using the case study method, this paper analyzes the social entrepreneurship process of 9 enterprises and the process mechanism of solving the rural poverty problem based on the identification and development of social opportunities. Our analysis suggests that social entrepreneurship is the process of identification, development and realization of social opportunities. Multidimensional rural poverty creates different social opportunities, including social opportunities in social, economic and ecological poverty. Enterprises integrate farmers into their value chain to develop and realize social opportunities, which is a sustainable means of poverty alleviation. In theory, we propose a conceptual framework for the sustainable development of social entrepreneurship and enriches the research on the process of realizing social opportunities in social entrepreneurship. In practice, we provide a sustainable development ideas for rural areas.

## Introduction

Rural poverty is the most prevalent type of human poverty in the world. Poverty alleviation in rural areas is a major global challenge. It is not only an economic issue but also a social issue related to inclusive development (Steiner and Teasdale, 2019). Traditional approaches to reducing rural poverty include government assistance, non-profit organization assistance, and corporate social responsibility. However, these approaches have problems, such as lack of capital, motivation and core competitiveness (Doherty et al., 2014). Therefore, how to deal with the shortage of external assistance and economic development in rural areas is still the key to reducing rural poverty. As an innovative way to solve social problems, social entrepreneurship plays an important role in solving the lack of external support and economic development difficulties in rural areas (Atahau et al., 2022).

Social entrepreneurship can integrate the efficiency, innovation and resources of traditional for-profit companies with the passion, values and mission of non-profit organizations, to identify and develop social opportunities based on social needs, thereby pursuing social, economic, and ecological values (Zulfiqar et al., 2021; Koehne et al., 2022). Rural areas are generally considered as the ideal location in which to build and operate social enterprises. Poverty here includes social, economic and ecological aspects (Khan et al., 2014; Liu et al., 2017), forming a variety of entrepreneurial opportunities (Alvarez and Barney, 2014). Then, how to identify and develop social opportunities to alleviate rural poverty is a challenge for social entrepreneurship.

However, the existing literature does not answer the above questions well. First, the mechanism and output of social enterprises in rural poverty alleviation remain ambiguous. As a rapidly developing academic field, some scholars have gradually begun to pay attention to the definition, value orientation and wider role of social entrepreneurship in solving social problems (Ranville and Barros, 2021). They argued that social entrepreneurship, which focuses on those at the bottom of the pyramid, is an effective way to address social problems such as poverty, uneven distribution of health resources and unemployment (Galaskiewicz and Barringer, 2012; McMullen and Warnick, 2016). However, social entrepreneurship in a rural context remains mostly unexplored (Steiner et al., 2021). Ghauri et al. (2014) found that social entrepreneurship is an effective way to eliminate poverty, but they were unable to clearly reveal its deep operating mechanism. Moreover, the sustainable way of solving problems by social entrepreneurship is worth exploring. Second, the types and realization processes of social opportunities in the context of rural poverty are still unclear. Opportunities have been widely discussed in the theoretical research of business entrepreneurship, but ignored in the field of social entrepreneurship (Davidsson, 2015). Effective opportunity identification is the premise of entrepreneurship, and opportunity development is the source of organizational competitive advantage. However, the existing research lacks systematic research on social opportunities in the context of rural poverty, and does not take into account the particularity of social entrepreneurship.

This research is guided by the following research question: How does social entrepreneurship solve rural poverty from the perspective of social opportunity? In answering this question, through literature review, we theoretically clarify the research status of social entrepreneurship and social opportunities in rural context. Then, we use case study method to explore the little-understood context of the process of social entrepreneurship (Yin, 2014). We analyze the process of identifying, developing and realizing the social opportunities of nine enterprises and reveal the mechanism of social entrepreneurship in the process of reducing rural poverty. In terms of identification of social opportunities, based on the sustainability theory, we refine the types of social opportunities from three dimensions: social poverty, economic poverty, and ecological poverty. In terms of the exploitation and realization of social opportunities, our study combines the value chain theory and explains the specific role of social entrepreneurship in rural poverty by revealing farmers’ value chain participation in the process of social entrepreneurship and the compatible ways of achieving social, economic and ecological benefits. We then propose an effective sustainable development framework for social entrepreneurship to promote the rural economy.

Our research contributes to entrepreneurship literature in two important ways. First, we enrich the research of social entrepreneurship from process perspective, and provide effective ways for social entrepreneurship to solve the problems of rural poverty. Second, we systematically study the types and realization process of social opportunities, which plays an important role in promoting the boundary expansion of entrepreneurship theory.

## Literature review

### Rural poverty and social entrepreneurship

Since 1980, poverty has been on the agenda of major international organizations (such as the United Nations, the World Bank, the International Monetary Fund). Narrowing the gap between urban and rural areas, eliminating extreme poverty, and achieving common prosperity are the ideals that human beings are constantly pursuing. In recent years, farmers have been forced to adapt to new challenges, such as market changes (Lans et al., 2013), information technology and biotechnology development, but rural poverty has not been adequately addressed (Rodriguez-Pose and Hardy, 2015). Poverty was initially considered to be an economic phenomenon, in which individuals or households were unable to meet basic living standards. Gradually, scholars have discovered that poverty is a multidimensional concept (Liu et al., 2017). Rural poverty is mainly discussed from three aspects of society, economy and ecology (Namara et al., 2010; Khan et al., 2014; Liu et al., 2017). Specifically, rural poverty issues include social exclusion, poor access to services and infrastructure, vulnerability to natural disasters, and an aging population caused by the migration of young people (Namara et al., 2010; Farmer et al., 2011; Alkire and Fang, 2019).

However, the actions of governments, commercial enterprises, and non-profit organizations often fail to effectively solve such problems (Ganapati and Reddick, 2018; Li et al., 2018); this has become known as a “triple failure” problem. Social entrepreneurship is an activity that maintains its operations by selling products or services in an innovative way, based on a clear social goal. It takes into account the efficiency, innovation and resources of business entrepreneurship, as well as the enthusiasm, values and mission of non-profit organizations, in order to provide innovative solutions for social poverty (Austin et al., 2006; Neck et al., 2009) and help communities meet complex social, economic and environmental challenges (Steiner and Teasdale, 2019).

To be sure, social entrepreneurship has a positive impact on rural issues (Steinerowski and Steinerowska-Streb, 2012), but few articles focus on its role in the rural context. Most of the existing studies focus on the definition, influencing factors, performance, legitimacy and other aspects of social entrepreneurship (Janssen et al., 2018; Stirzaker et al., 2021; Chen et al., 2022). However, social entrepreneurship is a complex activity, and scholars have paid insufficient attention to its process. In terms of research context, the research focuses on the results of social entrepreneurship in solving a wide range of social problems. The research on the particularity of social entrepreneurship to solve rural problems is not deep enough. In addition, the goal of social entrepreneurship is to use appropriate capabilities to ensure economic success, positive environmental impacts and social benefits. That is, sustainable entrepreneurship pursues the triple bottom line of economic, social and ecological goals (Belz and Binder, 2017). However, due to its special nature between business and charity, it is worth thinking about how social entrepreneurship can solve rural poverty in a sustainable way.

### The role of social opportunity in social entrepreneurship

Social opportunity is an entrepreneurial opportunity in the context of social entrepreneurship. Entrepreneurial opportunity refers to the mismatch between the demand and the corresponding product or service supply, which is the core of business entrepreneurship and social entrepreneurship (Shane and Venkataraman, 2000; Mair and Marti, 2006; Davidsson, 2015). The identification, development and utilization of entrepreneurial opportunities is an important aspect of the entrepreneurial process, which is also applicable to the field of agricultural entrepreneurship (Lans et al., 2013; Belz and Binder, 2017). It provides an unsaturated market for products or services and requires innovation or improvement of existing products or services (Singh, 2001).

The meaning and function of opportunities are different in the two entrepreneurial contexts. However, scholars pay more attention to opportunities in the business field. In an organization with a business mission, the entrepreneurial opportunity is often considered an opportunity to make money, with market response at its core. Therefore, it is difficult to apply to opportunities in the context of social entrepreneurship (Corner and Ho, 2010; Lehner and Kansikas, 2012). There are social opportunities in social evils and social problems (Lumpkin et al., 2013). Entrepreneurs should comprehensively consider factors such as social and moral environments and recognize that social entrepreneurship is an effective way to solve social problems. It is important that business activities be legal and socially beneficial (Brooks, 2009). Opportunity identification in the context of social entrepreneurship, which reflects the entrepreneur’s ability to detect value creation (Perrini et al., 2010) and the entrepreneur’s willingness to solve these social problems (Lumpkin et al., 2013), is the starting point and core of the social entrepreneurship process. Unfortunately, social entrepreneurship is still a relatively new concept in the academic field, and the research on opportunity identification in the field of social entrepreneurship is relatively scattered and unsystematic. For example, some scholars focus on the opportunity identification behavior of youth when preparing for social entrepreneurship (Zulfiqar et al., 2021). Moreover, the research on the types and realization process of social opportunities in the rural context is insufficient; multi-dimensional rural poverty provides different social opportunities, which needs to be summarized.

## Research design

### Methods

Quantitative and qualitative research are the two basic research methods (Creswell and Creswell, 2017). Qualitative research is a practice-oriented method, especially the case study method. It can describe the phenomenon of things (cases) and analyze the reasons in detail according to the actual development of enterprises, which is conducive to excavating the general rules and constructing new theories. In the field of social entrepreneurship research, most studies use qualitative research methods. For example, Cherrier et al. (2018), based on the ethnographic case of social risk in India, studied the possibility of institutional complexity providing opportunities for social entrepreneurs and identified strategic countermeasures to deal with institutional complexity. Munoz and Kibler (2016) used the fuzzy set method to explore the relationship between institutional complexity and social entrepreneurship.

This paper adopts the case study method for the following three reasons. First of all, this paper mainly discusses the mechanism and process of social entrepreneurship to alleviate rural poverty, which is still in its initial stage. Compared with quantitative methods that are conducive to testing theories, the case study method is more suitable for answering “how” and “why,” which helps this research to complete theoretical construction (Yin, 2014). Second, there are multiple constructs such as social opportunities and social entrepreneurship, each of that contains multiple subdivided dimensions. The case study method can be used to describe the dimensions and the relations of different constructs in a detailed way, which is helpful to reveal the relationships hidden behind the evolving and complex phenomena. Third, social entrepreneurship is an effective way to solve social problems, but there is little mature theoretical guidance on how to reduce rural poverty. Case study is a more appropriate research method to explore contextualization, which can develop rural real-life cases into a conceptual framework supported by existing literature (Pervez et al., 2013). We can improve the reliability and validity of the study by using multi-case replication logic, and make the conclusion testability and empirical validity (Eisenhardt, 1989; Yin, 2014).

### Case selection and collection

Different from the statistical sampling principle in empirical studies, the selection of case study objects is mainly based on theoretical sampling (Glaser and Strauss, 1967), that is, the case selection should be consistent with the research theme, rather than representative of the whole. In this way, theoretical insights can be obtained through the connection between constructs (Eisenhardt and Graebner, 2007). This selection criterion based on case specificity rather than generality is known as “exploratory logic” (Yin, 2014).

Since research on social entrepreneurship is still in its infancy, given the research purpose, time, cost and difficulty of collection, there are three types of case sources: (1) case studies and papers, ensuring that their information is clear, accessible, and verified; (2) the official website of social entrepreneurial organizations, marketing materials and statistics provided by enterprises, and news reports; and (3) the website of the Trickle Out Africa Project and Business Call to Action (BCtA). Trickle Out provides an open case study platform for users, researchers and decision makers, and its public information comprises data on nearly 4,000 companies in 19 countries; the BCtA website provides a database of high-quality, inclusive business models across sectors and regions in 70 countries.

After screening, this paper identified a total 9 representative cases of rural social entrepreneurship, such as Nuru Energy, Drishtee and Tekera Resource Centre (Table 1). These cases come from various industries (agriculture, medical, education, energy, tourism, etc.) and countries (China, India, Bangladesh, etc.). Compared with homogeneous enterprises, heterogeneous enterprises provide a more solid theoretical foundation and improve the external validity of the research (Santos and Eisenhardt, 2009).

**TABLE 1:** Cases of rural social entrepreneurship.

Case	Country	Founder	Date of establishment
Xingeng workshop	China	Zhu Bingzhao	2006
Drishtee	India	Nitin Gachhayat	2000
Tekera resource center	Uganda	Brigitte	2006
Acceso El Salvador	El Salvador	Clinton Giustra Enterprise Partnership	2013
Bancalimentos	Colombia	Olga Bocarejo	2015
CD finance	China	Liu Dongwen	2008
Grameen Veolia Water	Bangladesh	Muhammad Yunus	2008
Njobvu cultural village lodge	Malawi	Several Villagers	2002
Fargreen	Vietnam	TrangTran	2015

### Coding and analysis

After data collection and collation, the research drew lessons from Corbin and Strauss’s (2008) grounded theory coding method and used the software Nvivo to code and analyze the cases. New concepts and ideas are abstracted from the data and logical argumentation is carried out under the idea of verification or falsification (Jantunen and Gause, 2014).

The steps are as follows: (1) Open coding. Frist, we coded the cases from A to I (e.g., Xingeng Workshop-A, Drishtee-B), and conceptualized the information content. Then, after 130 initial concepts were obtained, they were combined and eliminated preliminarily to obtain 101 valid concepts. Finally, the concept was categorized to form 23 conceptual sub-categories. (2) Axial coding. This paper analyzed the potential relationships between the sub-categories and gradually integrated the main categories. (3) Selective coding. The research summarized the main categories as core categories or theoretical dimensions, and systematically associate them with other categories, thereby constructing a systematic theoretical framework. When coding, we constantly compared, analyzed and modified categories with similarities and differences, so as to improve theoretical accuracy and realize theoretical innovation (Kroeger et al., 2014). Due to the complexity of the coding process, refer to Ausrød et al. (2017), the research only shows the coding results, as shown in Table 2. Moreover, there are many first-order codes, so we have listed the typical concepts and the number of items.

**TABLE 2:** Data coding and analysis.

Typical concepts (no. of items)	Conceptual sub-categories	Conceptual categories	Aggregated theoretical dimensions
Lack of equal employment opportunities (4)	Job creation	Social opportunities	Rural social opportunity
Lack of necessary skills; low level of education (3)	Education service	in social poverty	
Limited (or no) basic medical services (6)	Medical service		
Limited access to markets for agricultural products (4)	Fair trade	Social opportunities	
Food insecurity; Lack cheap and safe supplies (2)	Low-price service	in economic poverty	
Few pledges; high agricultural risks (4)	Microcredit		
Lack of ecological planting technology (4)	Ecological technology	Social opportunities	
Inadequate utilization of ecological resources (5)	Ecological resource	in ecological poverty	
Waste recycling; straw burning pollution (5)	Ecological protection		
Buy their crops; optimize quality management (3)	Farmers participate in procurement link	Farmers as suppliers	Value chain participation
Make handicrafts; make reusable bags (3)	Farmers participate in manufacturing link	Farmers as	
Sell handicrafts; provide a retail platform (3)	Farmers participate in marketing link	employees	
Tour guide; technical guidance (3)	Farmers participate in service link		
Provide convenient medical services (5)	Farmers as consumers	Farmers as consumers	
Public welfare consumption; cross-subsidy (5)	Profit model	Economic benefit	Sustainable social
Product development; production (3)	Profitable products		entrepreneurship
Activity income; service charges (4)	Profitable services		
Expansion of trade; expansion of service network (3)	Market expansion		
Youth education Fund; experience sharing platform (4)	Provide high-quality education services	Social benefit	
Build employment platform; create jobs (2)	Create local employment opportunities		
Skills training; to meet the demand for skilled labor (2)	Rural human capital development		
Improve income; return to social life (6)	Improve the quality of life		
Health education seminar; affordable medical expenses (4)	Improve medical conditions		
Reduce emissions; green production (4)	Improve the ecological environment	Ecological benefit	
Carry out a series of lectures on ecology (3)	Promote ecological education activities		
Reshape rural charm; promote local culture (4)	Develop rural resources and local culture		
Convert organic waste into organic fertilizer (3)	Strengthen waste management		

### Explanation of core constructs

Based on the existing literature, this paper selected and clarified the measure methods that best match the case data, so that the core constructs emerged from the cases. Their definition and explanation are as follows:

#### Rural social opportunity

The rural social opportunity is the social opportunity in the rural context. The essence of social entrepreneurship is the process of identifying, exploiting and realizing social opportunities. With the rapid development of the global economy and the modernization of agriculture, rural development and construction have lagged far behind the demand for rural transformation, and social imbalances often coexist with unmet social needs.

There are still many poverty issues that have social, economic and ecological aspects (Namara et al., 2010; Khan et al., 2014; Liu et al., 2017), including low population density, isolated communities, a lack of large town centers, and a lack of effective public transportation and sound infrastructure (Steiner and Teasdale, 2019). These provide a large number of development opportunities for social enterprises (Littlewood and Holt, 2018; Steiner and Teasdale, 2019). Moreover, opportunities for entrepreneurship may differ according to various issues (Alvarez and Barney, 2014).

Drawing on the dimensions of rural poverty and multidimensional poverty assessment methods (Bourguignon and Chakravarty, 2003; Khan et al., 2014), the research summarized three types of rural social opportunities in social, economic and ecological poverty, including job creation, education service, medical service, fair trade, low-price service, microcredit, ecological technology, ecological resource and ecological protection.

#### Value chain participation

The identification and development of opportunities seems to be related to the active participation of stakeholders and the mobilization of resources (McDermott et al., 2018). Studies have shown that although the economic development in rural areas is terrible (Pateman, 2011), when people believe that inequity is great or the pain is severe, they are more inclined to act quickly, and the resulting community cohesion has prompted a high level of trust and active citizen participation in rural communities. In the process of developing social opportunities, more and more social enterprises have developed a collaborative approach between service users and providers to meet existing challenges (Boyle and Harris, 2009), including farmers in their enterprise value chains.

The enterprise value chain includes the process of obtaining raw materials from the original supplier until the final product is delivered to the user (Shank and Govindarajan, 1993). The participation of farmers can be divided into three types: as suppliers participating in the enterprise’s procurement link, as employees participating in the manufacturing, marketing and service links, or as consumers of the enterprise.

First, social enterprises establish supply and marketing cooperative relationships with farmers, purchase their products directly, and build convenient, smooth, efficient, and stable circulation channels and docking platforms between the agricultural product market and the market (Barrett et al., 2012) to return more income to farmers. Second, allowing social enterprises to participate in the manufacturing, marketing and service links means that farmers are included as employees in the workforce and thus can directly participate in the daily operations of the enterprise. This can reduce social isolation (Steiner and Teasdale, 2019) and promote the employment of rural surplus labor, which is obviously a win-win strategy. This requires companies to be able to transform their values from instrumentalists into values that include equality and social justice (Tobin et al., 2016). Furthermore, in modern society it is no longer possible for farmers to be completely self-sufficient, and every aspect of life requires one to purchase goods and receive services from business operators. Social enterprises regard farmers as customers at the end of the value chain, provide farmers with better services, popularize technology, and disseminate knowledge to meet their urgent needs in terms of spiritual, material, and cultural aspects.

#### Sustainable social entrepreneurship

Social enterprise, which integrates the elements of business and charity (Austin et al., 2006; Mair and Marti, 2006), is an ideal hybrid type of organization that combines aspects of multiple organizational forms. Therefore, the challenge for social enterprise is to balance their mixed goals, i.e., achieving sustainable commercial development, meeting the needs of “transactional” customers, and achieving social goals. With conflicting goals, hybrid enterprises may struggle to achieve financial sustainability, and research is called to reconcile these conflicting goals. According to the theory of sustainable development, sustainable rural social entrepreneurship should identify, develop and utilize opportunities to provide goods and services with social, economic and ecological benefits (Belz and Binder, 2017). In particular, with regard to economic sustainability, enterprises have different sources of income, i.e., providing high-quality services, which can reduce their dependence on national funds and other donations, and it is more conducive to independent sustainable development.

### Reliability and validity

In order to ensure the reliability and validity, the following measures were taken in this study: (1) The reliability and validity of research design. This study follows the reproducibility principle of multiple case studies (Yin, 2014) to compare and verify the research conclusions, thus enhancing the persuasiveness. (2) The reliability and validity of case selection. The nine social enterprises belong to different regions and industries, which helps ensuring that information covers a certain theoretical breadth, and improving the scalability and external validity of research design. It is conducive to compare whether there are differences in the exploitation and realization of social opportunities in different poverty circumstances, so as to enhance the external validity of the research conclusions. (3) Reliability and validity of data collection. The case database was established to incorporate data from different sources for triangulation verification, so as to form an accurate and complete data chain. (4) Reliability and validity of data encoding. The researcher first determined the coding standard, then coded the first case, adjusted the coding rules after comparison, and finally coded the eight cases to ensure the uniformity of the coding standard. (5) Theory construction. After the theoretical dimensions were initially determined, other social enterprises were selected for the theoretical saturation test. By encoding and analyzing this part of data in turn, the extracted categories and main categories have been included in the existing categories, and no new categories have been extracted. This showed that the main category was well developed, and its structural dimension had a good theoretical saturation, so the sampling was stopped.

## Results

### Identification, exploitation and realization of social opportunity

#### The process of identifying social opportunity

##### Social opportunities in social poverty

Rural social poverty is an unfair condition, a phenomenon caused by the imbalanced distribution of resources between urban and rural areas, low levels of farmers’ knowledge and skills, and loss of health (Khan et al., 2014; Liu et al., 2017). In this situation, three types of social opportunities have been created: job creation, education service, and medical service.

Farmers are often socially excluded because of their low levels of education and lack of necessary skills (Munoz and Steinerowski, 2012). This provides an educational service-oriented opportunity for social enterprises to realize the development of human capital for farmers and reduce the unequal opportunities stemming from differences in personal background and living conditions, so that all people can enjoy equal dignity and the ability to live (Nussbaum, 2009). In addition, the community is always looking for new strategies and income sources, that is, developing new non-agricultural income-generating activities on their farms (Alsos et al., 2011), hoping to increase local employment opportunities for young people and reduce their outward migration (Steiner and Teasdale, 2019). This provides social enterprises with job creation opportunities, replacing traditional charity subsidies with farmers finding work, allowing them to rely on their own labor force to obtain a secure income and realize their self-worth. Furthermore, disease is currently an important cause of rural poverty (Liu et al., 2017), while rural towns and villages have limited (or no) basic medical services. Most rural medical problems involve a lack of chronic disease care, a shortage of health workers, the failure to adequately address prevention issues, a lack of infrastructure for comprehensive care, etc. (Humphreys and Wakerman, 2008). Therefore, medical service-oriented social opportunities inspire social enterprises to provide farmers with affordable and high-quality medical services.

##### Social opportunities in economic poverty

Rural economic poverty usually means that farmers do not have a stable income and cannot meet their basic consumption needs. Poverty can be reduced by increasing agricultural income or reducing expenditures (Banerjee et al., 2015; Koch, 2015). This creates three social opportunities for social entrepreneurship: fair trade, low-price service, and microcredit social opportunities. There are limited opportunities for agricultural products to enter the market (Perez et al., 2013), and their purchase prices are volatile (Dethier and Effenberger, 2012). However, farmers often lack the ability to cope optimally with agricultural production and trading activities. Fair trade opportunities encourage social enterprises to establish supply and marketing partnerships with the poor, provide vulnerable farmers with a stable and fair source of income, and protect them from market fluctuations. In addition, due to remote geographical locations and low consumption levels, rural commodity markets are small and fragmented, and middlemen are asking high prices from rural consumers, which often prevents rural households from obtaining enough product information (Zaefarian et al., 2015) or buying the goods they need from a more competitive (low-price) market (Vachani and Smith, 2008). There is a greater demand for affordable basic necessities and services in rural areas, which in turn provides social enterprises with low-price, service-oriented social opportunities. Furthermore, for rural families, limited funding is a key obstacle (Duong and Izumida, 2002; Duong and Thanh, 2014). However, farmers are often excluded from the trajectory of financial institutions due to low pledges, high agricultural risks, the high lending costs of financial institutions, and low credit records, resulting in serious asymmetry between financial services and financial needs in rural areas. This offers a microcredit-type social opportunity to provide villagers with personal or commercial loans at a reasonable interest rate.

##### Social opportunities in ecological poverty

Poor natural conditions in rural areas (Namara et al., 2010), coupled with an irrational use of resources, environmental pollution and other human activities, often lead to ecological poverty. This in turn gives rise to three types of social opportunities: ecological technology, ecological resource, and ecological protection.

First, rural areas lack technologies related to clean energy and waste disposal (Chauhan and Saini, 2015). Eco-technological social opportunity requires enterprises to solve a series of rural problems scientifically and efficiently using advanced technological means. Second, one of the causes of rural poverty is the inadequate utilization of rural ecological and cultural resources. The diversification of traditional agriculture into non-agricultural enterprises is an important corporate strategy (Dias et al., 2019). Relying on agricultural production, developing agricultural resources and the local culture by means of tourism is an effective means of sustainable agricultural development (Gao and Wu, 2017), one that provides opportunities for social enterprises to develop ecological resources. Third, rural environmental pollution is one of the main problems hindering rural development. Pollution comes from waste discharged during agricultural production, such as livestock manure, plant straw, wood chips, straw, and residual pesticides (Pindado and Sanchez, 2017). This serious problem provides social enterprises with opportunities for ecological protection, which can support the natural environment by protecting local land and fully protecting biodiversity (Steiner and Teasdale, 2019) to promote the application of a circular economy and sustainable agricultural development.

#### The process of exploitation and realization social opportunity

##### How does social entrepreneurship solve the problem of “social poverty”?

The problem of social poverty has created social opportunities for job creation, education services, and medical services. Taking Xingeng Workshop as an example, the founder realized that giving money could not permanently alleviate poverty. The company produces specialty handicrafts and brings farmers into the sales chain to obtain economic and social benefits. In addition, they create ecological value by recycling Tetra Pak packaging materials, recycling environmentally friendly products and conducting training courses on ecological education and rural development.

##### How does social entrepreneurship solve the problem of “economic poverty”?

The issue of economic poverty has led to fair trade, low-price service, and microcredit social opportunities. A typical example of identifying and exploiting low-price service-oriented social opportunities is Bancalimentos. The company created a circular economy, acquired organic waste and recyclable materials, sold them as raw materials to the local recycling industry, bought large quantities of food, medicines and other household items at economic returns, and sold them to villagers at affordable prices. As a result, they indirectly achieve the purpose of increasing the income of the poor while reducing environmental waste pollution.

##### How does social entrepreneurship solve the problem of “ecological poverty”?

The problem of ecological poverty gives rise to the social opportunities of ecological technology, ecological resource development and ecological protection. A typical example of identifying and developing ecological resource-development social opportunities is Njobvu Cultural Village Lodge. They hire local villagers to participate in the service link of the value chain and carry out interesting Malawian cultural activities. While enjoying high-quality accommodation services, tourists can observe traditional pastimes such as dancing, cooking, and basket weaving. Through this project, tourism development has provided a source of income for villagers and directly improved orphan care, local schools, clinics and bridges. It has also reduced poaching in Liwonde National Park, which encourages communities to protect this precious natural resource.

**FIGURE 1:**
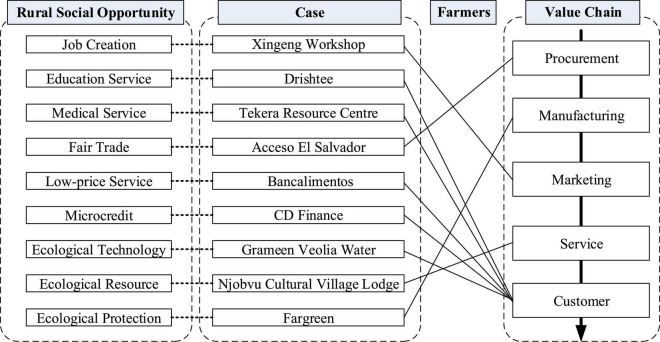
The internal mechanism of social entrepreneurship to solve rural problems.

Through grounded theoretical analysis of 9 cases, the research explored the internal mechanism of social entrepreneurship to solve rural problems (Figure 1); that is, by identifying and developing social opportunities, social enterprises include farmers in their value chains, allowing them to participate in the procurement, manufacturing, marketing and service or consumer’s links. This allows enterprises to create social value, economic value and ecological value in order to solve the problem of rural poverty.

In fact, the best way to help poor farmers is not to donate money, goods or other free assistance directly to them, as traditional poverty-alleviation subjects do, as this may generate spiritual poverty. In contrast, social enterprises use the means of integrating farmers into the entire social value chain to ensure that farmers can create social, economic, and ecological values with dignity through their labor and intelligence. In addition, the development of poverty-alleviation value chains as a poverty-reduction strategy can be used to counter the failure of institutions such as the government (Thorpe, 2018). This is the best way to truly benefit the livelihood of small farmers.

### Sustainable development framework for social entrepreneurship

**FIGURE 2:**
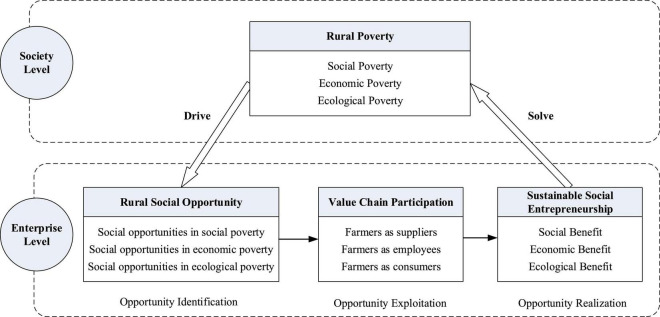
Sustainable development framework for social entrepreneurship.

Through the generalization and reasoning of the internal mechanism of social entrepreneurship to solve the problem of rural poverty, and taking into account the constraints of second-hand data and geographical location, this study summarizes the conceptual framework of sustainable development for social entrepreneurship rather than utilizing an empirical model (Figure 2).

Sustainable social entrepreneurship is the process of identifying, developing, and utilizing opportunities. The goods or services they provide have social, economic, and ecological benefits, which is in line with the triple bottom line principle (Belz and Binder, 2017). The entire process of social entrepreneurship includes the impact of the two levels of society and enterprise, which is in line with the multilevel attributes of social enterprises (Le Pennec and Raufflet, 2018).

At the social level, multidimensional rural poverty often puts farmers in a difficult position, and they lack resources and skills. Compared with other groups, they are more likely to fall into the intergenerational poverty cycle (Lichter et al., 2015). However, many poverty issues coexist with the urgent needs of villagers, generating numerous development opportunities waiting to be discovered by social enterprises.

At the enterprise level, when a social enterprise recognizes a social opportunity, it often takes a series of actions to creatively use and combine resources to meet social needs (Mair and Marti, 2006). We find that in the process of solving rural poverty, the strategic action taken by social enterprises is to integrate farmers as suppliers, employees, and target customers into the value chain of the enterprise, and create social, economic and ecological value with (or for) them (Ebrahim et al., 2014; Dohrmann et al., 2015; Saebi et al., 2019).

This is a sustainable way of solving the problem of rural poverty, that is, to solve problems at the social level as the guideline and to take the strategy of the enterprise level as the promotion point. Social enterprises include farmers in the value chain, mobilize people to actively participate in poverty alleviation, and combine rural external and internal resources to improve rural predicaments in education, employment, medical care, and green energy. This will have long-term rather than short-term positive impacts on many aspects of economy, society and ecology, and in the end fulfill the mission of solving rural poverty.

## Discussion

This paper explores the contribution of social entrepreneurship to rural poverty alleviation from the perspective of social opportunity. We analyze the process of social entrepreneurship based on the identification, development and realization of social opportunities. We then summarize the types of social opportunities, the ways in which addressing rural poverty works, and the resulting social, economic and ecological outcomes.

First, our research enriches the social entrepreneurship theory from process perspective, clarifies the connotation of social opportunities and reveals the realization process of social opportunity and its special value in social entrepreneurship. Social opportunities arise from three types of poverty: social poverty, economic poverty, and ecological poverty. Based on these factors, we summarize nine typical social opportunities in rural poverty. There are human capital, property rights, and financial capital that can be exploited in different types of social opportunities. If entrepreneurs are unaware of the potential for value creation in various opportunities, their effectiveness in participating in poverty initiatives may be limited (Alvarez and Barney, 2014). In terms of opportunity development and realization, we introduce the theory of enterprise value chain and believe that farmers’ participation in different value chain links is the primary means of realizing social opportunities. Companies can use their expertise to develop affordable products or services to address the unmet needs of the poor (Zaefarian et al., 2015), or empower them by treating them as suppliers, producers or consumers of the company (Boyle and Boguslaw, 2007). This finding highlights the importance of exploitation of social opportunities in the entrepreneurial process and also responds to the call of scholars to study opportunities in rural areas (Tabares et al., 2022). Based on the value chain theory, we make the complex approach of poverty alleviation more actionable. In addition, we can clearly show that social entrepreneurship may have several goals when solving problems. For example, Xingeng Workshop has the dual goals of promoting farmers’ employment and protecting the rural ecological environment. Consistent with traditional entrepreneurial theory centered on opportunities (Shane and Venkataraman, 2000; Zahra et al., 2008), we believe that the discovery and development of opportunities are crucial to any research work related to new business concepts, and we must find answers by studying entrepreneurial opportunities. However, we also believe that in the context of social entrepreneurship, opportunity is special valuable (Zulfiqar et al., 2021), which determines that the core of social entrepreneurship is social value creation rather than economic value. Therefore, our findings extend the research paradigm of social entrepreneurship beyond the framework of business entrepreneurship, and we believe it can contribute to this emerging research field.

Second, we analyze the mechanism of social entrepreneurship to solve rural poverty, and fill in the research gap of rural context in the field of social entrepreneurship. Most entrepreneurship research has an urban focus (Tabares et al., 2022), and the social entrepreneurship literature has also largely ignored rural entrepreneurial activities, especially in underdeveloped countries, where theoretical and empirical studies are still limited. Our study therefore focuses on the countryside and finds that rural social entrepreneurship plays a key role in alleviating extreme poverty. Social entrepreneurship can integrate both social and entrepreneurial dimensions, and social opportunity is the primary medium and focus of poverty. At the social level, one must focus on difficult social issues and grasp the urgent needs of people at the bottom of the pyramid (Goyal et al., 2015). At the enterprise level, social enterprises must establish clear social goals (such as improving education and health, reducing social exclusion, etc.), engage in business activities in innovative ways, and maintain their operations by selling products or services (Galaskiewicz and Barringer, 2012; McMullen and Warnick, 2016). These are two aspects of social enterprises’ sustainable solution to social problems. During the implementation process from the social to the enterprise level, social enterprises must begin by identifying social opportunities. By identifying and developing social opportunities, the social level and enterprise level can be combined to focus on specific rural poverty problems, so that solutions can be implemented and poverty problems solved. This double-sided research complements existing social entrepreneurship research and helps to further understand how social entrepreneurship is integrated with rural poverty or other social issues.

Third, we have constructed a sustainable development framework for social entrepreneurship aimed at helping to find a sustainable solution to rural poverty. From a sustainable livelihood perspective, the framework proposes a multi-dimensional measurement approach with the goal of improving the livelihoods of vulnerable individuals and communities in rural areas. We argue that sustainable livelihoods are multi-dimensional, as poverty can be manifested in many ways and affected by many factors, not just income (Tabares et al., 2022). Therefore, social entrepreneurship needs to take into account social, economic and ecological benefits. Traditional poverty-reduction methods often assume that the poor cannot help themselves and need charity, and so direct public investment, subsidies, or other charities are used to meet unmet needs; however, this impact is often limited and short-term (Austin et al., 2006). On the other hand, the market-based approach recognizes that poverty does not necessarily eliminate one’s participation in business and market transactions (Zaefarian et al., 2015). In fact, in order to meet their basic needs, individuals must trade with cash or labor. Therefore, in rural areas, compared with other helping entities, social enterprises see farmers as suppliers, employers, and consumers, which seems to better help communities control and address complex social, economic, and environmental challenges (Steiner and Teasdale, 2019). This can fill the gap between what the private sector is willing to produce and what the government and charity can provide, and it is an effective mechanism for creating value for (or with) farmers (Saebi et al., 2019). This also helps to solve the triple failure problem of government, non-profit organizations and commercial enterprises, and fundamentally promotes the development of entrepreneurship theory.

## Conclusion, implication and limitations

### Conclusion

This study uses a case study method to analyze the identification, development and realization of social opportunities in the process of social entrepreneurship under the rural context. We try to reveal the mechanism of social entrepreneurship to solve the rural poverty, and propose a conceptual framework for the sustainable development of social entrepreneurship. We find that social entrepreneurship is a process of identifying, developing and realizing social opportunities, and the economic value, social value and ecological value created by social entrepreneurship correspond to the solution of rural economic, social and ecological poverty. This is the essential process of social entrepreneurship promoting rural development. We also find the role of social opportunity in addressing rural poverty at both the social and corporate levels. There are three types of social opportunities driven by rural poverty at the social level, including opportunities in social, economic and ecological poverty. At the enterprise level, after identifying social opportunities, enterprises engage farmers in different parts of their value chain to develop and realize opportunities, which is a sustainable means of addressing poverty.

### Implication

This study is of great significance both theoretically and practically for social entrepreneurship in solving the rural poverty. Firstly, this paper extends the theoretical research on the process perspective in the field of social entrepreneurship and answers how promoting poverty alleviation in rural areas. This study integrates rural poverty issues at the social level with actions at the enterprise level, fills the gap of social entrepreneurship theory in the rural field. From the perspective of social opportunities, we put forward the sustainable development framework of social entrepreneurship, which complements and improves the sustainability of social entrepreneurship. In practice, this paper provides concrete and sustainable ideas for solving rural poverty through social entrepreneurship. In addition, it has certain guiding significance to solve the problem of insufficient external support from the government, commercial enterprises and non-profit organizations.

Secondly, this paper enriches the research on the realization processes of social opportunities in the rural context. At present, the research on opportunity recognition in the field of social entrepreneurship is scattered. Moreover, the existing research on social opportunities focuses on the research paradigm of commercial enterprises and ignores the particularity of social opportunities. We summarize the rural social opportunities in social poverty, economic poverty and ecological poverty. It provides ideas for enterprises to identify social opportunities effectively, and also fills the gap of research. Also, we find that farmers’ participation in the value chain is an important means of social opportunity development. It not only helps to explain the mechanism process of social entrepreneurship to solve rural poverty, but also helps to guide the practice of social entrepreneurship, and provides a new solution path for enterprises to realize social, economic and ecological value. The introduction of value chain lines also helps to visualize solutions to the complex problem of rural poverty. By taking farmers as suppliers, employers and consumers, social entrepreneurship not only neatly solves the obstacles to the sustainable development, but also helps rural areas to fundamentally control and deal with complex social challenges.

### Limitations

While our study offers some important insights, it also has limitations that open the way for future research. First, our research limits the sources of social opportunities to the three dimensions of poverty, and there are further sources and types of social opportunities waiting to be explored. Second, due to time and resource constraints, our study is limited to a conceptual framework rather than utilizing an empirical model. Nonetheless, we believe that theoretical generalizations of the mechanisms emerging in this study are possible. Future research can use multi-source data such as interviews and panel data to conduct more rigorous empirical tests and develop it into a successful model. In addition, the universality of the model remains to be further examined in different contexts. Future research could focus on a certain region or country and propose more targeted poverty solutions.

## Data availability statement

The raw data supporting the conclusions of this article will be made available by the authors, without undue reservation.

## Author contributions

XZ: designing. YS and YG: writing. YD: method. All authors contributed to the article and approved the submitted version.
